# Identification of “safe harbor” loci in indica rice genome by harnessing the property of zinc-finger nucleases to induce DNA damage and repair

**DOI:** 10.3389/fpls.2014.00302

**Published:** 2014-06-26

**Authors:** Christian Cantos, Perigio Francisco, Kurniawan R. Trijatmiko, Inez Slamet-Loedin, Prabhjit K. Chadha-Mohanty

**Affiliations:** ^1^Gene Transformation Lab, Plant Breeding, Genetics, and Biotechnology Division, International Rice Research InstituteMetro Manila, Philippines; ^2^Indonesian Center for Agricultural Biotechnology and Genetic Resources Research and DevelopmentBogor, Indonesia

**Keywords:** zinc-finger nucleases (ZFNs), safe harbor loci, rice (*Oryza sativa* L.), homologous recombination (HR), double-strand breaks (DSBs)

## Abstract

Zinc-finger nucleases (ZFNs) have proved to be successful tools for targeted genome manipulation in several organisms. Their main property is the induction of double-strand breaks (DSBs) at specific sites, which are further repaired through homologous recombination (HR) or non-homologous end joining (NHEJ). However, for the appropriate integration of genes at specific chromosomal locations, proper sites for gene integration need to be identified. These regions, hereby named safe harbor loci, must be localized in non-coding regions and possess high gene expression. In the present study, three different ZFN constructs (pZFN1, pZFN2, pZFN3), harboring β-glucuronidase (GUS) as a reporter gene, were used to identify safe harbor loci on rice chromosomes. The constructs were delivered into IR64 rice by using an improved *Agrobacterium*-mediated transformation protocol, based on the use of immature embryos. Gene expression was measured by histochemical GUS activity and the flanking regions were determined through thermal-asymmetric interlaced polymerase chain reaction (TAIL PCR). Following sequencing, 28 regions were identified as putative sites for safe integration, but only one was localized in a non-coding region and also possessed high GUS expression. These findings have significant applicability to create crops with new and valuable traits, since the site can be subsequently used to stably introduce one or more genes in a targeted manner.

## Introduction

Rice (*Oryza sativa* L.) has emerged as a model cereal system for molecular studies as the complete genome has been sequenced, several tools for functional genomics are available, and the production of transgenic plants by efficient *Agrobacterium*-mediated transformation is easier than with other major cereals (Izawa and Shimamoto, 1996). In addition, rice is one of the leading food crops worldwide and increasing rice production is expected to play a significant role in reducing hunger and upgrading the economic status of developing countries.

Nowadays, due to recent advances in molecular biology, research focuses more and more on the ability to manipulate genomes at specific sites. Efficient methods for genome editing further promote gene discovery and functional gene analyses in model plants as well as the introduction of novel desired agricultural traits in important species. Genome editing technology enables efficient and precise genetic modification through the induction of a double-strand break (DSB) in a specific target sequence, followed by the generation of desired modifications during the subsequent DNA break repair (Puchta, 2002). Genome editing is achieved by integrating desired DNA molecules into the target genome by employing mainly the homologous recombination (HR) pathway. However, in plants, these molecules are normally delivered by direct gene-transfer methods and often integrate into the target cell genome via non-homologous end joining (NHEJ) instead of HR (Britt and May, 2003). Currently, zinc-finger nucleases (ZFNs), transcription activator-like effector nucleases (TALENs), and clustered regulatory interspaced short palindromic repeats (CRISPR)/Cas-based RNA-guided DNA endonucleases are used as innovative techniques in genome editing (Gaj et al., 2013). These nucleases diverge in different aspects, starting from the composition, to specificity and mutation signatures (Kim and Kim, 2014). Knowledge of their specific features is essential for choosing the most appropriate tool for a range of applications.

ZFNs were among the first tools used for genome editing a decade ago, and are defined as artificial restriction enzymes composed of a fusion between the DNA-binding domain of a zinc-finger protein (ZFP) and the cleavage domain of the FokI endonuclease. The DNA-binding domain of ZFPs can be engineered to recognize a variety of DNA sequences, while the FokI endonuclease domain, which functions as a dimer, cleaves the DNA and creates DSBs (Durai et al., 2005; Porteus and Carroll, 2005). Through directed co-localization and dimerization of two FokI nuclease monomers, ZFNs generate a functional site-specific endonuclease that creates a DSB at the targeted locus (Mani et al., 2005). Through the use of this methodology, the induced DNA sequence modifications can range from mutations to gene replacement, site-specific structural changes, or gene insertion, to name a few (Husaini et al., 2011).

DNA repair of DSBs is primarily carried out through HR and NHEJ. Depending on the desired modification, either pathway can be used in ZFN-mediated genomic engineering. Since HR relies on homologous DNA to repair the DSB, gene targeting can be achieved by supplying an exogenous template, termed a donor sequence, which is replicated and mostly used to introduce small mutations or large insertions. On the other hand, NHEJ is an error-prone repair process, ideal for generating mutations that can result in gene knockouts or knockdowns when the ZFN-mediated DSB is introduced into the protein coding sequence of a gene (Bibikova et al., 2003; Urnov et al., 2010). ZFNs have been successfully used for inducing DSBs in the genomes of various species, including plants (Lloyd et al., 2005; Wright et al., 2005). Successful HR-based gene replacement was observed at frequencies ranging from 0.2 to 4% in tobacco protoplasts, where acetolactase synthase genes *SurA* and *SurB* were mutated to confer resistance to herbicides (Townsend et al., 2009). In maize, Shukla et al. (2009) showed that insertional disruption of the *IPK1* gene, encoding the inositol-1,3,4,5,6-pentakisphosphate enzyme, resulted in both herbicide tolerance and alteration of the inositol phosphate profile in developing seeds. In addition, the trait/modification was stably transmitted to the next generation.

For the successful integration of genes at specific chromosomal locations, it is of utmost importance to identify proper sites for gene insertion. The results of Day et al. (2000) have shown that a transgene can be delivered into a specific chromosome position; this will allow the selection of a specific target site for a consistent and higher transgene expression. Therefore, the ability to achieve site-specific manipulation of the rice genome can improve the expression of transgenes as it is highly dependent on the locus of integration. These integration regions must possess high gene expression and preferably be localized in non-coding DNA regions (Curtin et al., 2012; Sadelain et al., 2012). In the present study, ZFNs were employed in order to identify such regions, hereby designated as safe harbor loci, on rice chromosomes. Three different ZFN constructs, containing β-glucuronidase (GUS) as a reporter gene, were used. The level of gene expression in different loci was measured through GUS assay, while the flanking regions were determined through thermal-asymmetric interlaced polymerase chain reaction (TAIL PCR). This represents the first report on the potential use of ZFNs for the identification of safe harbor loci in plants. A number of important agronomic traits to improve rice for higher yield, tolerance of environmental stresses, and metabolic engineering are polygenic in nature. A large number of genes are needed to modify the metabolic pathway; the safe harbor loci will allow pyramiding of transgenes in one locus. The results presented here can be of great practical applicability in generating crops with improved agronomic traits.

## Results

### Generation of transgenic rice plants using ZFN constructs

**Figure 1:**
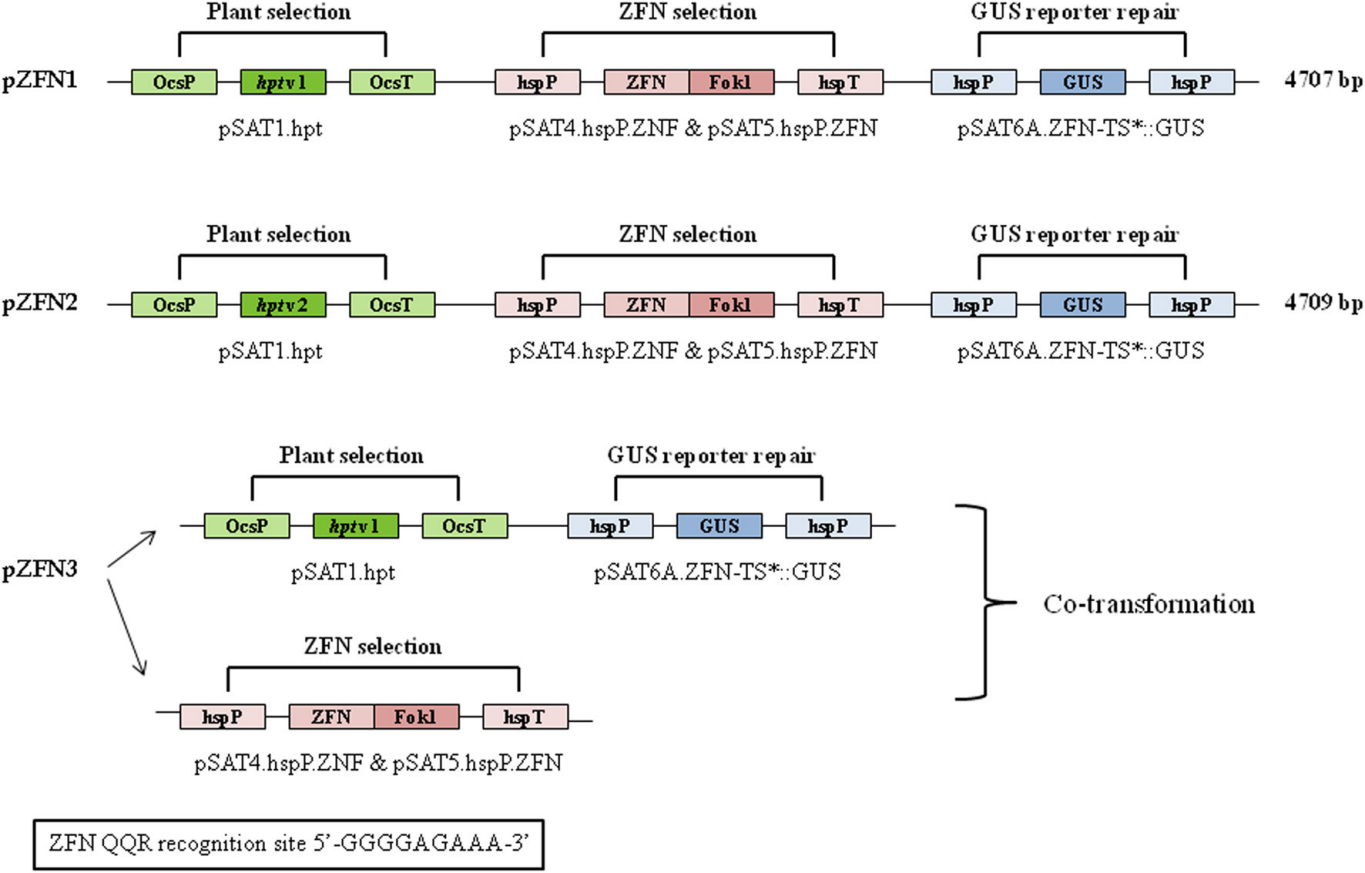
**Structure and key features of the pZFN1, pZFN2, and pZFN3 constructs**. The vector system is based on the assembly of ZFN expression cassettes (pSAT4.hspP.ZFN and pSAT5.hspP.ZFN), a plant selection expression cassette (pSAT1.hpt), and GUS reporter cassette (pSAT6A.ZFN-TS^*^::GUS) onto the plant binary vector pRCS2. Asterisk stands for the modification generated in the cassette. The plasmid carries a plant expression cassette engineered for constitutive expression of a mutated uidA (GUS) gene. A stop (TGA) codon was engineered within the 6-bp spacer of the ZFN target site, leading to premature termination of uidA translation in plant cells. The ZFN QQR recognition site is shown.

**Figure 2:**
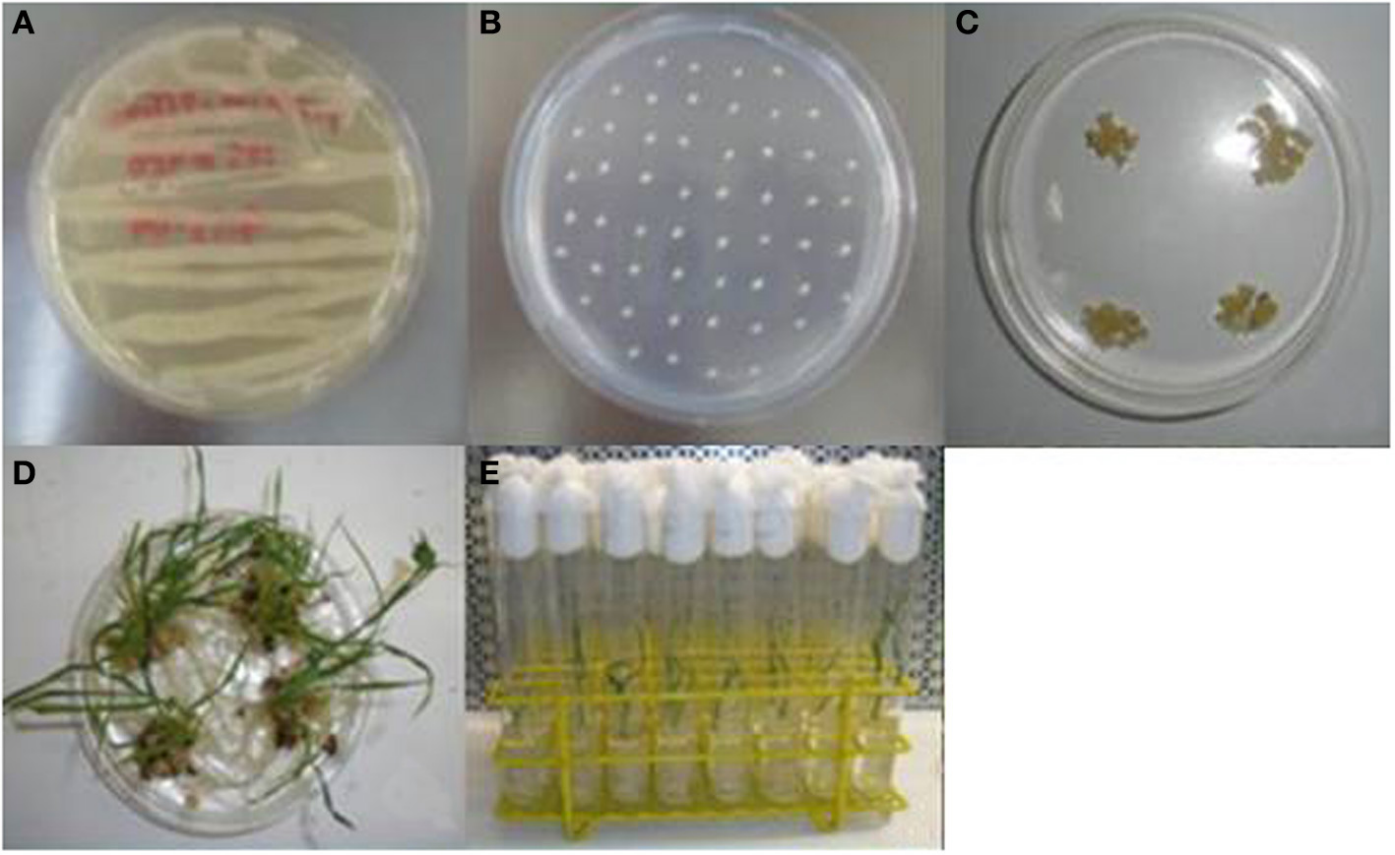
**Generation of transgenic rice plants by *Agrobacterium*-mediated transformation. (A)** Growth of *A. tumefaciens* LBA4404 on AB medium; **(B)** co-cultivation of immature embryos with *Agrobacterium* suspension; **(C)** selection of resistant calli; **(D)** regeneration of plantlets; **(E)** rice plantlets on MS0 rooting media.

Three different constructs, pZFN1, pZFN2, and pZFN3, were used to generate transgenic rice plants. The vector system is based on the assembly of ZFN expression cassettes, a plant selection expression cassette, and a GUS reporter cassette, onto the plant binary vector pRCS2. The constructs contain the *hpt* (hygromycin phosphotransferase) gene driven by the octopine synthase promoter (OcsP), while the GUS and ZFN expressions are driven by a heat-shock inducible promoter (hspP; GenBank Acc. No. NC_003076.8). The difference between pZFN1 and pZFN2 consists of the length of the *hpt* gene. Each construct was introduced into *A. tumefaciens* LBA4404 and subsequently co-cultivated with rice immature embryos. In the case of pZNF3, co-transformation of two binary vectors, one carrying the plant selection marker and the reporter repair plasmid and the other carrying only the constitutive ZFN expression cassette, was used (Figure 1). *Agrobacterium*-mediated transformation steps are summarized in Figure 2. Constructs pZFN1 and pZFN2 generated 171 (85.5%) and 133 (88.5%) calli resistant to hygromycin, while the pZNF3 construct showed the highest number of resistant calli (439, 146.3%). From the regenerable callus culture, 29 GUS-positive plants were obtained for pZFN1, 60 for pZFN2, and 188 for pZFN3 (Table 1). Based on the number of immature embryos used and the plants obtained, transformation efficiency was calculated for each construct. Results are shown in Table 1. The highest transformation efficiency (66.3%) was registered when the pZFN3 construct was used.

**Table 1:** **Transformation efficiency of embryogenic calli derived from immature embryos of IR64 rice infected with *A. tumefaciens* LBA4404 containing ZFN constructs**.

**Constructs**	**Immature embryos**	**Resistant calli (%)**	**Regenerated calli (%)**	**GUS-positive plants**	**Transformation efficiency (%)**
pZFN1	200	85.5	32.2	29	27.5
pZFN2	150	88.7	49.6	60	44.0
pZFN3	300	146.3	45.3	188	66.3

### GUS expression levels in rice transgenics

**Figure 3:**
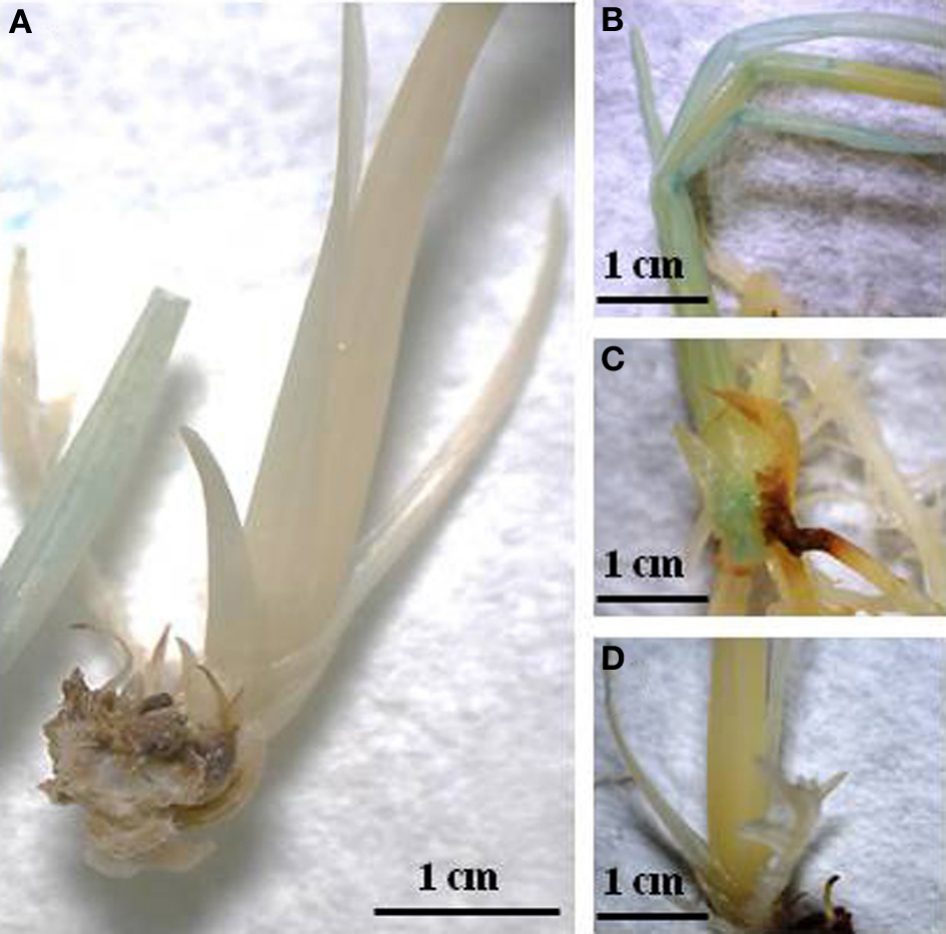
**Histochemical GUS staining; (A) Wild type; (B) Transgenic rice exhibiting high GUS expression; (C) Transgenic rice exhibiting medium GUS expression; (D) Transgenic rice exhibiting low GUS expression**.

Following *Agrobacterium*-mediated transformation, rice-positive transformants were identified based on histochemical GUS detection. Two-week-old plantlets were initially incubated at 42°C for 90–150 min in order to trigger expression of the GUS gene, which is driven by a hspP. Using the Image J software, pixel density is measured based on the blue color present in plant tissue. The numerical values obtained, were then categorized in three different levels of GUS intensity (high, medium, and low). Figure 3 shows the histochemical GUS analysis. The number of positive events categorized accordingly with the levels of GUS expression is presented in Table 2. Out of 29 positive events obtained using pZFN1, 8 showed high expression, while 21 events showed low GUS expression. In the case of pZNF2, out of 60 positive events, only 1 presented high GUS expression, 8 showed medium expression, and 51 showed low expression. The highest number of events with high GUS expression (113) was obtained when the pZFN3 construct was used.

**Table 2:** **Events positive for GUS integration separated into groups (high, medium, and low) based on the intensity of GUS staining expression**.

**Constructs**	**Total events**	**High GUS expression**	**Medium GUS expression**	**Low GUS expression**
pZFN1	29	8	0	21
pZFN2	60	1	8	51
pZFN3	188	113	40	30

### Identification of flanking sequences and safe harbor loci regions

**Figure 4:**
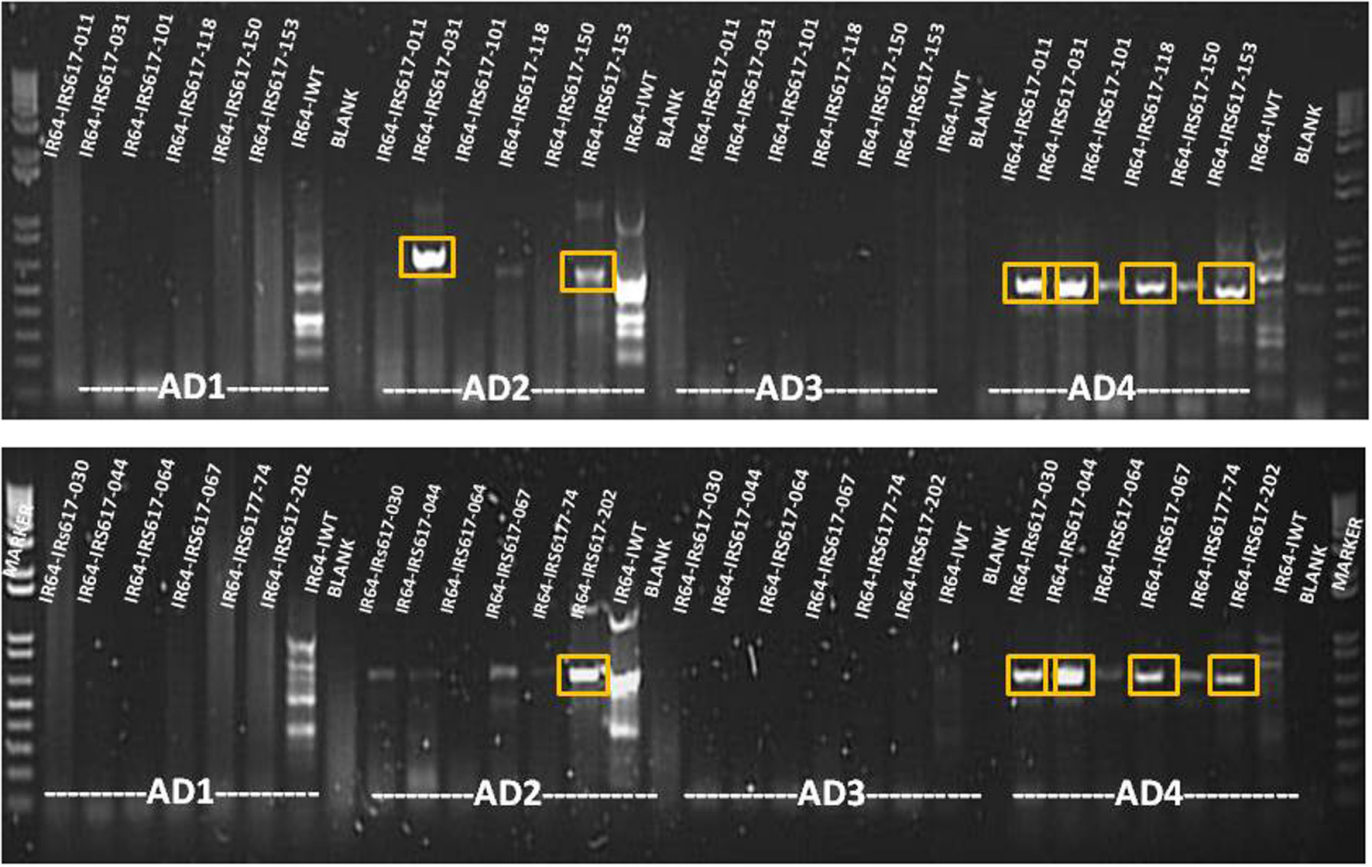
**Agarose gel analysis of TAIL PCR products amplified from GUS-positive insertion lines**. Bands shown in boxes were cut and sequenced. AD1, AD2, AD3, and AD4 = non-specific primers.

Genomic DNA was extracted from all positive events and a two-step TAIL PCR was performed in order to identify the flanking sequences (Figure 4). All GUS-positive plants with different levels of expression gave specific TAIL-PCR products ranging from 500 to 1000 bp. Bands were subsequently purified from gel, cloned and sequenced. Following bioinformatic analysis, 28 sites for GUS insertion were identified (Supplementary Table 1). However, some of these regions were too short to be considered as safe harbor loci, while others presented low GUS expression. Out of the putative sites identified, three sequences chosen from plants exhibiting high GUS expression also presented a proper size (Table 3). One event showed integration on chromosome 1 (3404275–3405012), three independent events presented integration on chromosome 8 (5490900–5491654), and four independent events were integrated on chromosome 3 (8499895–8500138). When the sequences were verified for the presence/absence of coding genes, the BLAST results showed that the region on chromosome 1 is part of a gene (LOC_Os01g07212) encoding a putative staphylococcal nuclease homolog. Similarly, the region located on chromosome 8 is part of a gene (LOC_Os08g09480) coding for OsFBX268, an F-box domain-containing protein. Only the locus on chromosome 3 was shown to be localized in a non-coding DNA region (Table 3). Two putative genes, LOC_Os03g15470 and LOC_Os03g15480, are located near this region (Supplementary Figure 1), but the 243-bp sequence on chromosome 3 (8499895–8500138) is considered as non-coding. Since this region was identified from plants with high GUS expression, and no putative coding gene, it can be considered as a safe harbor locus for gene insertion. The nucleotide sequence was also converted to amino acid sequence, and no putative protein was shown to be encoded in this region.

**Table 3:** **Chromosomal localization and annotation of putative safe harbor loci identified in transgenic rice plants obtained by using ZFN constructs**.

**Sample ID**	**GUS expression**	**Chromosome region**	**Sequence length (bp)**	**Gene annotation**	**Protein**
IR64-IRS617-176	High	Chr3:8499895–8500138	243	Non-coding	Non-coding
IR64-IRS617-177	High	Chr3:8499895–8500138	243	Non-coding	Non-coding
IR64-IRS617-190	High	Chr3:8499895–8500138	243	Non-coding	Non-coding
IR64-IRS617-202	High	Chr3:8499895–8500138	243	Non-coding	Non-coding
IR64-IRS617-201	High	Chr1:3404275–3405012	737	LOC_Os01g07212	Staphylococcal nuclease
IR64-IRS617-026	High	Chr8:5490900–5491654	754	LOC_Os08g09480	F-box domain
IR64-IRS617-030	High	Chr8:5490900–5491654	754	LOC_Os08g09480	F-box domain
IR64-IRS617-087	High	Chr8:5490900–5491654	754	LOC_Os08g09480	F-box domain

## Discussion

In the present study, the ZFNs characteristic of inducing a DSB and subsequently trigger a response of proper DNA-repair pathways, was used to successfully insert the β-glucuronidase marker gene into the rice genome with the purpose of identifying safe regions for gene integration. The originality of this work derives from the use of ZFNs as an innovative technique for plant genome editing, associated with a newly standardized protocol for rice *Agrobacterium*-mediated transformation. Furthermore, the final result, identification of safe harbor loci for gene insertion, has a great impact related to practical applications in agriculture.

Several strategies can be used for ZFNs to modify the genome of plant species, depending on the presence and structure of the donor DNA and the plant DNA-repair machinery. Targeting a specific genomic sequence requires the delivery and expression of two ZFN monomers in the same cell, with the final goal of inducing site-specific mutagenesis, gene stacking, and/or gene replacement (Urnov et al., 2010).

Three constructs carrying the semi-palindromic target site of QQR ZFN were employed to generate transgenic rice plants. QQR (Glutamine-Glutamine-Arginine) ZFN is a well-defined three-finger ZFN capable of recognizing and binding to 5'-GGGGAAGAA-3' nucleotide sequence. It was among the first chimera nucleases used and since then was successfully applied for genome engineering in both animals and plants (Smith et al., 2000; Weinthal et al., 2013). The vectors are based on the structure of a previously described pSAT plant expression vector system specifically designed to facilitate the assembly of multi-gene expression cassettes (Tzfira et al., 2005). The expression of the QQR coding sequence, as well as the expression of the GUS gene, is controlled by a hspP. This type of construct was successfully used in generating targeted mutations in *Arabidopsis* plants (Lloyd et al., 2005). The authors also estimated that QQR, when expressed under a heat-shock promoter, can induce mutations at a frequency as high as 0.2 mutations per gene, which is considerably higher than the previously reported HR-dependent frequencies (from 10^−7^ to 10^−4^) for plant cells (Iida and Terada, 2005).

Among the three different constructs used for plant transformation, pZNF3 showed the highest transformation efficiency (66.3%) as well as the highest number of GUS-positive events (188 plants). This finding suggests that co-transformation of two separate vectors (one carrying the plant selection marker and the reporter repair plasmid, and the other carrying only the constitutive ZFN expression cassette) is more efficient than when the ZFNs and donors are delivered together in the same construct. In addition, the pZFN2 construct possessing a shorter variant of *hpt* gene showed higher transformation efficiency (44%) than pZFN1 (27.5%).

One of the main goals of genetic engineering is to attain stable transgenic events possessing predictable and reproducible levels of expression that can be characterized in terms of the effect and implications of transgene insertion. In order to achieve this, it is important to gather detailed information about the transgene insertion site. The main attribute for safe regions to be used as gene integration sites is localization in non-coding DNA regions that permit high gene expression (Curtin et al., 2012; Sadelain et al., 2012). Techniques used for this purpose include detection of the physical position of transgenes by fluorescent *in situ* hybridization (FISH) (Salvo-Garrido et al., 2001; Choi et al., 2002) as well as genetic map position (Salvo-Garrido et al., 2004). Information on transgene insertions can also be obtained from the analysis of flanking regions (Sha et al., 2004). To identify the insertion site of the T-DNA flanking sequence, analyses were conducted using TAIL PCR. The method consists of the use of nested T-DNA border region-specific primers together with shorter arbitrary degenerate primers for the unknown genomic DNA region flanking the insertion site (Liu et al., 1995). Such priming creates both specific and non-specific products, whose relative amplification efficiencies can be thermally controlled. In two serial PCRs, the unspecific products are gradually diluted out and in the final reaction the specific products are detectable on the gel by a slight shift in size due to the nested priming in the T-DNA region. In the present study, 28 flanking sequences for the GUS gene were identified following rice transformation with ZFN constructs. However, most of these sites possessed low expression and/or were localized in DNA coding regions. Only one region, located on chromosome 3 (8499895–8500138), retained both attributes and it can be considered as a safe harbor locus for gene insertion. This finding is highly important for future practical applications that could lead to the creation of crops with new valuable traits. In addition, ZFNs as part of new plant breeding technologies allow genome editing without the introduction of foreign DNA; thus, the resulting crops could be classified as non-GMO (Pauwels et al., 2014).

## Materials and methods

### Vector design

The constructs used in the present study were assembled and validated as per Tovkach et al. (2010). A schematic representation of plasmid maps is shown in Figure 1. The vector system is based on the assembly of ZFN expression cassettes (based on pSAT4.hspP.ZNF and pSAT5.hspP.ZNF plasmids), a plant selection expression cassette (based on pSAT1.hpt plasmid), and a GUS reporter cassette (based on pSAT6A.ZFN-TS^*^::GUS plasmid) onto the plant binary vector pRCS2. The nucleotide sequences of pSAT1.hpt, pSAT6A.ZFN-TS^*^::GUS, pSAT4.hspP.ZNF, and pSAT5.hspP.ZNF plasmids are available on the NCBI site. The plant selection cassette contains the *hpt* gene, which confers resistance to hygromycin and is driven by the OcsP. The GUS and ZFN expressions are driven by a hspP (GenBank Acc. No. NC_003076.8). The GUS reporter cassette contains a mutated GUS reporter gene, engineered to carry a TGA (stop) codon within the 6-bp spacer of the ZFN target site, constructed on a reporter repair plasmid. The QQR ZFN recognition site is 5'-GGGGAAGAA-3'. Three different constructs were used and they were designated as pZFN1, pZFN2, and pZFN3 (Figure 1). The difference between pZFN1 and pZFN2 consists in the length of the *hpt* gene. As for pZNF3, it requires co-transformation of two binary vectors: one carrying the plant selection marker and the reporter repair plasmid and the other carrying only the constitutive ZFN expression cassette.

### *Agrobacterium*-mediated transformation and plant regeneration

*Agrobacterium*-mediated transformation of IR64 rice (*Oryza sativa* L. *indica*) was performed as described by Slamet-Loedin et al. (2014). Immature embryos (IE) harvested from rice panicles at 8–12 days after anthesis were dehulled and sterilized. IE were co-cultivated with *A. tumefaciens* LBA4404 for 7 days at 25°C under dark conditions in A201 medium. Following co-cultivation, IE were transferred onto sterile Petri dishes containing A202 selection medium complemented with hygromycin and incubated under continuous light at 30°C for 5 days (first selection). Subsequently, IEs were placed on A203 medium and incubated under the same conditions for 10 days (second selection). After this period, the embryogenic calli were selected and placed on A203 medium under the same conditions and time period (third selection).

For plant regeneration, the resistant calli were incubated on pre-regeneration medium (A204) under continuous light at 30°C for 10 days. Proliferating calli were selected and transferred onto regeneration medium (A205). Subsequently, individual regenerated plantlets were placed in sterile glass tubes containing MS0 rooting medium and kept under continuous light at 25°C for 14 days. Plant material was then used for further analysis.

### Histochemical GUS assay

The location of GUS activity in plant tissues was determined histochemically as described by Jefferson et al. (1987). The GUS reaction mix consisted of the following: 50 mmol/L potassium ferrocyanide, 50 mmol/L potassium ferricyanide, 5 mL 0.2 mol/L sodium phosphate buffer, 0.5 mol/L sodium EDTA and 10% Triton X-100, and water. A separate solution of X-Gluc (5-Bromo-4-Chloro-3-Indolyl-Beta-D-Glucuronide) (Biosynth, Switzerland) at a concentration of 25 mg X-Gluc/mL of N-N dimethyl formamide was added to this reaction mix at a ratio of 352 μ L of reaction mix to 48 μ L of X-Gluc solution. In order to trigger the expression of the GUS gene, 2-week-old rice plantlets were incubated for 90–150 min at 42°C and subsequently recovered for an additional 24–72 h prior to GUS staining. Plantlets were then incubated in the GUS solution at 37°C for 16 h. The GUS solution was discarded and the plantlets were rinsed with water and bleached sequentially with 25, 50, and 75% ethyl alcohol and finally kept in 95% ethyl alcohol. Quantification of GUS activity was performed by using ImageJ (v. 1.45) software to identify the intensity of GUS gene expression and accordingly the transgenics were labeled as having high, medium, and low expression.

### Genomic DNA extraction

Rice genomic DNA was extracted and purified following the protocol described by Dellaporta et al. (1983) with some modification in the extraction buffer as follows: 1 M Tris-HCl (pH = 8.0), 0.5 M EDTA, and 4 M NaCl and sodium bisulfate. Following extraction, genomic DNA was measured spectrophotometrically (NanoDrop ND-1000, NanoDrop, USA) by UV absorption at 260 nm, while DNA purity was evaluated on the basis of the UV absorption ratio at 260/280 nm and analyzed by 1% agarose gel electrophoresis in 1 × TAE SYBR SAFE® (Invitrogen, USA) staining.

### Thermal-asymmetric interlaced polymerase chain reaction (TAIL PCR)

TAIL PCR was performed using the 5-Prime Taq DNA polymerase (5-Prime, USA) in a G-STORM® PCR System (Somerton Biotechnology Centre, UK) as per the supplier's recommendation. Genomic DNA isolated from plants with different levels of GUS expression was subjected to two separate PCR runs. Primer sequences are shown in Supplementary Table 2. The reaction mix for the primary amplification was prepared in a total volume of 20 μ L and contained 1 × 5-Prime Taq DNA polymerase buffer, 1 U 5-Prime Taq DNA polymerase, 0.2 mM dNTPs, 0.2 μ M specific left border primer (LB_pRCS2_F1), and 3 μ M of each arbitrary degenerate primer (AD1, AD2, AD3, and AD4). Each arbitrary degenerate primer was paired to the specific left border primer, resulting in four primer pair reactions for each sample. The PCR program for the primary TAIL PCR included 5 cycles at 94°C for 1 min, 55°C for 1 min, and 72°C for 2.3 min, 1 cycle at 94°C for 30 s, 44 °C for 1 min, and 72°C for 2.3 min, 15 cycles at 94°C for 30 s, 55°C for 1 min, and 72°C for 2.3 min, and the last cycle at 94°C for 30 s, 44°C for 1 min, and 72°C for 2.3 min, with a final elongation step at 72°C for 5 min. After the primary TAIL PCR, amplification products were diluted 10× for the secondary amplification. The reaction mixture contained the same components as the primary PCR, except for a different specific left border primer (LB_pRCS2_F2). The program included 20 cycles at 94°C for 30 s, 55°C for 1 min, 72°C for 2.3 min, 94°C for 30 s, 44°C for 1 min, and 72°C for 2.3 min, followed by a final elongation step at 72°C for 10 min.

All TAIL PCR products were visualized on a 1.5% agarose gel. PCR products were excised and purified using a QIAquick gel extraction kit (Qiagen, USA). The purified products were ligated in a pGEM T-easy vector system (Invitrogen, USA) and transformed in *E. coli* DH5a strain using the heat-shock method. Following bacterial transformation, the white/blue screening method was used to identify the positive colonies, which were then grown in LB liquid medium overnight at 37°C. Plasmid DNA was extracted using a Purelink® Quick Plasmid Miniprep kit (Invitrogen, USA) and digested using *EcoRI* restriction enzyme (Invitrogen, USA) to check for the presence of the ligated PCR product. Subsequently, plasmids were sequenced and analyzed with bioinformatic tools (Macrogen, Korea).

### Bioinformatic analysis

NCBI BLASTn (http://blast.ncbi.nlm.nih.gov/Blast.cgi) was used to locate the sequenced regions on the corresponding chromosomes. Sequences were checked using the rice annotation sites MSU (http://rice.plantbiology.msu.edu), RAP-DB (http://rapdb.dna.affrc.go.jp/), and Gramene (www.gramene.org/). Nucleotide sequences were transcribed to proteins using Expasy Tools (http://web.expasy.org/translate/) and the putative protein sequences were verified using the UniProt database (www.uniprot.org/uniprot/).

### Conflict of interest statement

The authors declare that the research was conducted in the absence of any commercial or financial relationships that could be construed as a potential conflict of interest.
