# Supplementary material for: Identification of “safe harbor” loci in indica rice genome by harnessing the property of zinc-finger nucleases to induce DNA damage and repair
Source: Front Plant Sci. 2014 Jun 26;5:302. doi: 10.3389/fpls.2014.00302 (PMC4071976; doi:10.3389/fpls.2014.00302)

**SUPPLEMENTARY MATERIAL**

**Supplementary Table 1.** Chromosomal localization and annotation of putative safe locus regions identified in transgenic rice plants obtained by using ZFN constructs.

| **Sample ID** | **Chromosome region** | **Gene annotation** | **Protein** | **Expression** |
| --- | --- | --- | --- | --- |
| IR64-IRS617-167 | Chr10:16198378-16198407 | Non-coding | Non-coding | None |
|  | Chr10:2283218-2283243 | Non-coding | Non-coding | None |
|  | Chr10:4965001-4965026 | Non-coding | Non-coding | None |
|  | Chr10:14350939-14350919 | Non-coding | Non-coding | None |
| IR64-IRS617-175-1 | Chr11:9163685-9163706 | LOC_Os11g16540 | Tetratricopeptide repeat domain- containing protein | Expressed |
|  | Chr11:15460398-15460418 | LOC_Os11g26910 | SKP1-like protein 1B | Expressed |
| IR64-IRS617-175-2 | Chr10:24955502-24955469 | Non-coding | Non-coding | None |
| IR64-IRS617-176-1 | Chr3:2283243-2283218 | Non-coding | Non-coding | None |
|  | Chr3:4965001-4965026 | Non-coding | Non-coding | None |
|  | Chr3:13553565-13553584 | Non-coding | Non-coding | None |
| IR64-IRS617-176-2 | Chr3:8499895-8500138 | Non-coding | Non-coding | None |
|  | Chr3:2666619-2666627 | Non-coding | Non-coding | None |
|  | Chr3:33550846-33550935 | LOC_Os03g58920 | Galactosyltransferase family protein | Expressed |
|  | Chr3:9547973-9548064 | LOC_Os03g17164 | Kinesin-related protein | Expressed |
|  | Chr3:25146860-25146944 | Non-coding | Non-coding | None |
| IR64-IRS617-177 | Chr3:12697454-12697482 | LOC_Os03g22160 | ReIA-SpoT like protein RSH1 | Expressed |
|  | Chr3:2283243-2283218 | Non-coding | Non-coding | None |
|  | Chr3:4965001-4965026 | Non-coding | Non-coding | None |
| IR64-IRS617-190-4 | Chr8:22730989-22731018 | Non-coding | Non-coding | None |
| IR64-IRS617-190-2 | Chr3:8499897-8500138 | Non-coding | Non-coding | None |
|  | Chr3:2666619-2666627 | Non-coding | Non-coding | None |
|  | Chr3:33550846-33550935 | LOC_Os03g58920 | Galactosyltransferase family protein | Expressed |
|  | Chr3:954767-9548064 | LOC_Os03g17164 | Kinesin-related protein | Expressed |
|  | Chr3:2514686-25146944 | Non-coding | Non-coding | None |
| IR64-IRS617-193 | Chr4:17062867-17062887 | Non-coding | Non-coding | None |
|  | Chr4:8293924-8293940 | Non-coding | Non-coding | None |
|  | Chr4:16415726-16415742 | Non-coding | Non-coding | None |
| IR64-IRS617-201-1 | Chr1:3404275-3405012 | LOC_Os01g07212 | Staphylococcal nuclease homologue | Expressed |
|  | Chr1:10366665-10366692 | LOC_Os01g18440 | sMADS89-MADS-box family | Expressed |
| IR64-IRS617-201-2 | Chr11:4960253-4960276 | Non-coding | Non-coding | None |
| IR64-IRS617-202 | Chr3:3404275-3405012 | Non-coding | Non-coding | None |
| IR64-IRS617-204 | Chr8:12483002-12483020 | Non-coding | Non-coding | None |
|  | Chr8:24181598-24181581 | Non-coding | Non-coding | None |
| IR64-IRS617-030 | Chr8:5490900-5491654 | LOC_Os08g09480 | OsFBX268 - F-box domain-containing protein | Expressed |
|  | Chr8:9961184-9961166 | Non-coding | Non-coding | None |
| IR64-IRS617-026 | Chr8:5490900-5491654 | LOC_Os08g09480 | OsFBX268 - F-box domain-containing protein | Expressed |
|  | Chr8:9961184-9961166 | Non-coding | Non-coding | None |
| IR64-IRS617-087 | Chr8:5490900-5491654 | LOC_Os08g09480 | OsFBX268 - F-box domain-containing protein | Expressed |
|  | Chr8:9961184-9961166 | Non-coding | Non-coding | None |

**Supplementary Table 2.** Oligonucleotide sequences used for thermal-asymmetric interlaced polymerase chain reaction (TAIL PCR).

| **Primer name** | **Sequence** | **Reference** |
| --- | --- | --- |
| LB-pRCS2_F1 | 5’-ATTCAATTCGGCGTTAATTCAGTACAT-3’ | current work |
| LB-pRCS2_F2 | 5’-AACGTCCGCAATGTGTTATTAAGTTGT-3’ | current work |
| LB-pRCS2_F3 | 5’-GTCCGCAATGTGTTATTAAGTTGTCTA-3’ | current work |
| LB-pRCS2_F4 | 5’-CGCAATGTGTTATTAAGTTGTCTAAGC-3’ | current work |
| AD1 | 5'-NTCGA(G/C)T(A/T)T(G/C)G(A/T)GTT-3' | Liu et al. 1995 |
| AD2 | 5'-NGTCGA(G/C)(A/T)-GANA(A/T)GAA-3' | Liu et al. 1995 |
| AD3 | 5'-(A/T)GTGNAG(A/T)ANCANAGA-3' | Liu et al. 1995 |
| AD4 | 5'-AG(A/T)GNAG(A/T)ANCA(A/T)AGG-3' | Liu et al. 1995 |

**Supplementary Figure 1.** Localization of putative safe locus regions on chromosomes 3, 1, and 8, as shown by the MSU Rice Genome Annotation Project (<http://rice.plantbiology.msu.edu/>).


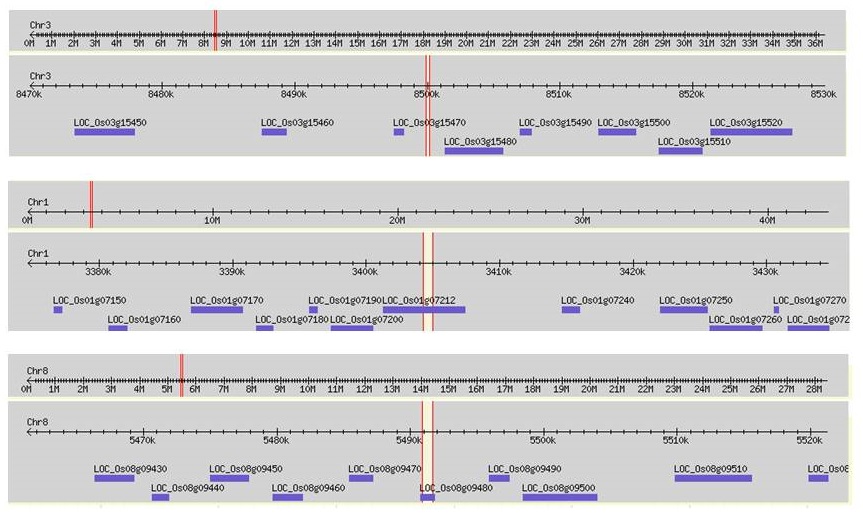

Supplement: Supplementary file 1 [file DataSheet1.DOCX]
